# Endoscopic endonasal dacryocystectomy

**DOI:** 10.1016/j.ajoc.2024.102244

**Published:** 2024-12-24

**Authors:** Juhi Daga, Mohammad Javed Ali

**Affiliations:** Govindram Seksaria Institute of Dacryology, L.V. Prasad Eye Institute, Hyderabad, India

**Keywords:** Lacrimal, Punctal agenesis, Dacryocystectomy, Endoscopic, Endonasal

## Abstract

**Purpose:**

To report an exceptionally rare instance of Endoscopic endonasal dacryocystectomy.

**Observations:**

Dacryocystectomy (DCT), a procedure of surgical extirpation of the lacrimal sac is normally approached by an external route. However, an endoscopic endonasal approach DCT is rare and usually reserved in cases where intellectual disabilities of a patient become a restrictive factor in maintenance of a healthy external wound. The present report describes a 14-years-old girl who presented with bilateral lacrimal sac swelling without epiphora. All the four puncta were absent. She was cosmetically concerned with the swellings and refused an external approach. The sac wall was grossly thin and cystic with remodelling of the bony lacrimal fossa which necessitated change of steps during an endoscopic DCT.

**Conclusion and importance:**

The present report describes a cosmetic indication and surgical technique of endoscopic endonasal dacryocystectomy in a young female with bilateral upper and lower punctal agenesis and slowly progressive congenital dacryocystocele without epiphora.

## 1 Introduction

Dacryocystectomy (DCT), a procedure of surgical extirpation of the lacrimal sac was described in a refined manner by John Thomas Woolhouse in 1724,^1^ although it was practiced in several crude ways since antiquity.^2^ The indications for DCT can be absolute (for example, lacrimal sac malignancy) or relative (for example, recurrent dacryocystitis in severe dry eyes).^3^ The approach for standard and extended DCT is external. However, an endoscopic endonasal approach DCT is rare and usually reserved for cases where intellectual disabilities of a patient become a restrictive factor in maintenance of a healthy external wound.^3^, ^4^, ^5^ Shams and Selva reported one such case where they performed an endoscopic endonasal DCT in an elderly patient with dementia.^5^ The present report describes another indication and surgical technique of endoscopic endonasal dacryocystectomy in a young female with bilateral upper and lower punctal agenesis and slowly progressive congenital dacryocystocele without epiphora.

## 2 Case report

**Fig. 1:**
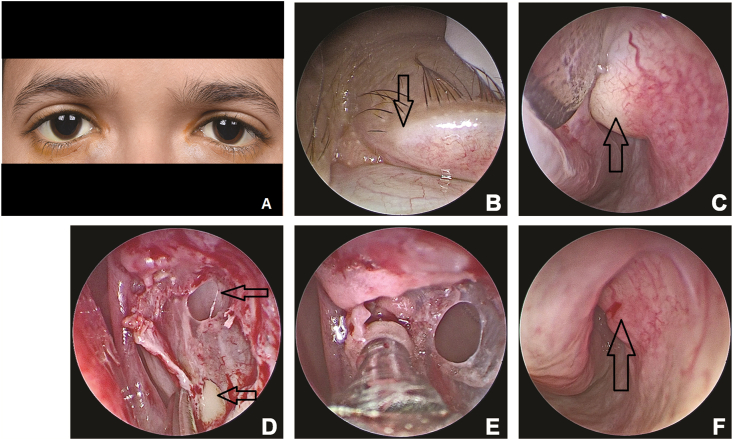
Clinical external image demonstrating bilateral congenital dacryocystoceles (Fig. 1A). Close up clinical image demonstrating punctal agenesis (black arrow, Fig. 1B). Endoscopic image of the left nasal cavity demonstrating the blind nasolacrimal duct dilatation in the inferior meatus (black arrow, Fig. 1C), thin lacrimal sac wall with buttonhole (black arrows, Fig. 1D), the thinned lacrimal sac wall (Fig. 1E), and the decompressed nasolacrimal duct (black arrow, Fig. 1F) Compare Fig. 1F with Fig. 1C.

The report adheres to the tenets of the declaration of Helsinki. Consent was obtained from the parents for identifiable photographs. A 14-year-old female presented with swelling in the corner of both eyes of 2-years duration. The swellings were progressively increasing in size and the patient was cosmetically concerned. (Fig. 1A). There was no history of epiphora or discharge. Upon examination there was a bilateral upper and lower punctal agenesis (Fig. 1B). The lacrimal sac swellings were below the medial canthal tendon and were bluish and cystic in appearance (Fig. 1A). An impression of bilateral upper and lower punctal agenesis with congenital dacryocystocele was made. Since the patient was asymptomatic in the context of epiphora, an option of dacryocystectomy was discussed. However, the patient was cosmetically concerned and refused an external approach procedure. Therefore, an endoscopic endonasal route was planned.

### 2.1 Surgical technique

**Fig. 2:**
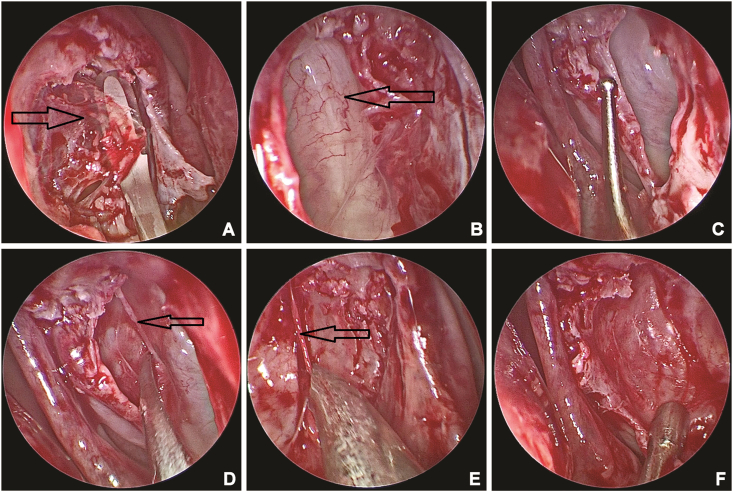
Endoscopic image of the right nasal cavity demonstrating the sac wall marsupialization. Note the membranous nature of the wall (Black arrow, Fig. 1A). Endoscopic image showing the interior of the lacrimal sacs showing the deep bony remodelling covered with a smooth lateral wall of the lacrimal sac with few vessels (Fig. 2B and C), and peeling of the walls from anterior and posterior ends (Black arrows, Fig. 2D and E). Picture at the end of the procedure (Fig. 2F).

Endoscopy guided bilateral lacrimal sac exposure and extirpation was performed under general anaesthesia. Intraoperative endoscopic evaluation revealed dilated and blind pouch of the nasolacrimal duct without any inferior meatal opening (Fig. 1C). Nasal decongestion, fashioning of the nasal flaps, and bony osteotomy was performed as per standard protocols of an endoscopic dacryocystorhinostomy (DCR).^6^ The lacrimal sacs were thin-walled and cystic (Fig. 1D and E) that were friable and could easily buttonhole on slight compression (Fig. 1E). Once a buttonhole happens, the sac contents empty and gets decompressed along with the nasolacrimal duct (Fig. 1F). The lacrimal sac marsupialization is completed as in a standard DCR (Fig. 2A). The bony lacrimal fossa is found to be enlarged and remodelled with thinned expanded lacrimal sac. The interior of the sac is unlike the normal sac, characterized by unremarkable smooth thin flimsy membrane with few vessels (Fig. 2B and C). The thin lacrimal sac walls were peeled off the bone from the anterior and posterior ends (Fig. 2D and E) to complete the dacryocystectomy procedure (Fig. 2F). Post-operative period was uneventful with complete resolution of the bilateral sac swellings and the patient was asymptomatic.

## 3 Discussion

Endoscopic endonasal dacryocystectomy was earlier described by Shams and Selva in a 78-year-old lady with dementia who had earlier undergone a DCR, which failed.^5^ There was a history of wound picking by the patient, dehiscence of the wound and subsequent infection. The surgery was like a standard endoscopic DCR till the stage of lacrimal sac marsupialization, following which the mobile lacrimal sac flaps and the posterior wall were removed using sharp through-biting straight Blakesley forceps.^5^ The common canalicular opening was cauterized and the nasal mucosa flap was reflected onto the lateral wall. The present case however did not have a well-developed lacrimal sac like the earlier case due to its congenital nature and different etiopathogenesis.

Delayed onset of dacryocystoceles in patients with upper and lower punctal agenesis without much epiphora is uncommon but have been reported earlier and was managed by marsupialization.^7^ The lacrimal sac walls are grossly thinned out (like in the present case) and histopathology examination demonstrate a single epithelial layer and poorly organized stroma.^8^ Cases with upper and lower punctal agenesis have been shown to also have concurrent canalicular agenesis and absent common canaliculus.^9^^,^^10^ Patients who are symptomatic in such scenarios can be managed by a CDCR with a Lester-Jones tube. However, the present case did not have epiphora and the reason for seeking medical advice was only a cosmetic concern secondary to bilateral dacryocystoceles. While there are several uncommon indications for performing a standard dacryocystectomy, the same is not true for an endonasal approach. The endoscopic approach is used in cases where the security of the external wound can be a threat from the patients themselves secondary to intellectual disabilities or mental illness.^3^, ^4^, ^5^ The present case adds significant cosmetic concern in a young female as an indication for an endonasal approach. The disadvantage of an endoscopic approach would be additional surgical involvement of the nasal mucosa and frontal process of maxilla. The present case being congenital in nature with less robust development of the lacrimal sac needed a simple peeling off its thinned walls from the bony lacrimal fossa to easily perform an endonasal dacryocystectomy. However, this may not be the case always, like the one presented by Shams and Selva.^5^ In situations where the lacrimal sac is well developed, one can perform the surgery as suggested by Shams and Selva.^5^ Can lacrimal sac marsupialization alone help? This is something that needs to be assessed in future cases with a longer follow up.

## CRediT authorship contribution statement

**Juhi Daga:** Writing – review & editing, Writing – original draft, Methodology, Data curation. **Mohammad Javed Ali:** Writing – review & editing, Writing – original draft, Supervision, Methodology, Conceptualization.

## Patient consent

Consent to publish this case report has been obtained from the patient(s) in writing. This report does not contain any personal identifying information.

## Financial disclosure

Mohammad Javed Ali receives royalties from Springer for the textbook “Principles and Practice of Lacrimal Surgery’ and Atlas “Atlas of Lacrimal Drainage Disorders” and the ‘Video Atlas of Lacrimal Drainage Surgery’.

## Authorship

All authors attest that they meet the current ICMJE criteria for Authorship.

## Funding

10.13039/501100005809Hyderabad Eye Research Foundation and JC Bose Grant of 10.13039/501100001843Science and Engineering Research Board of Govt of India (MJA).

## Declaration of competing interest

The authors declare that they have no known competing financial interests or personal relationships that could have appeared to influence the work reported in this paper.
